# Systematic large-scale assessment of the genetic architecture of left ventricular noncompaction reveals diverse etiologies

**DOI:** 10.1038/s41436-020-01049-x

**Published:** 2021-01-26

**Authors:** Francesco Mazzarotto, Megan H. Hawley, Matteo Beltrami, Leander Beekman, Antonio de Marvao, Kathryn A. McGurk, Ben Statton, Beatrice Boschi, Francesca Girolami, Angharad M. Roberts, Elisabeth M. Lodder, Mona Allouba, Soha Romeih, Yasmine Aguib, A. John Baksi, Antonis Pantazis, Sanjay K. Prasad, Elisabetta Cerbai, Magdi H. Yacoub, Declan P. O’Regan, Stuart A. Cook, James S. Ware, Birgit Funke, Iacopo Olivotto, Connie R. Bezzina, Paul J. R. Barton, Roddy Walsh

**Affiliations:** 1 Cardiomyopathy Unit, Careggi University Hospital, Florence, Italy; 2 Department of Experimental and Clinical Medicine, University of Florence, Florence, Italy; 3 National Heart and Lung Institute, Imperial College London, London, UK; 4 Cardiovascular Research Centre, Royal Brompton and Harefield NHS Foundation Trust, London, UK; 5 Laboratory for Molecular Medicine, Partners HealthCare Personalized Medicine, Cambridge, MA USA; 6 Amsterdam UMC, University of Amsterdam, Department of Clinical and Experimental Cardiology, Heart Centre, Amsterdam, Netherlands; 7 MRC London Institute of Medical Sciences, Imperial College London, London, UK; 8 Genetic Unit, Careggi University Hospital, Florence, Italy; 9 Department of Paediatric Cardiology, Meyer Children’s Hospital, Florence, Italy; 10 Aswan Heart Centre, Aswan, Egypt; 11 Department of Neurosciences, Psychology, Drug Research and Child Health (NeuroFarBa), University of Florence, Florence, Italy; 12 National Heart and Lung Institute, Heart Science Centre, Harefield Hospital, London, UK; 13 National Heart Research Institute Singapore, National Heart Centre, Singapore, Singapore; 14 Cardiovascular and Metabolic Disorders Program, Duke-National University of Singapore, Singapore, Singapore; 15 Department of Pathology, Harvard Medical School/Massachusetts General Hospital, Boston, MA USA

## Abstract

**Purpose:**

To characterize the genetic architecture of left ventricular noncompaction (LVNC) and investigate the extent to which it may represent a distinct pathology or a secondary phenotype associated with other cardiac diseases.

**Methods:**

We performed rare variant association analysis with 840 LVNC cases and 125,748 gnomAD population controls, and compared results to similar analyses on dilated cardiomyopathy (DCM) and hypertrophic cardiomyopathy (HCM).

**Results:**

We observed substantial genetic overlap indicating that LVNC often represents a phenotypic variation of DCM or HCM. In contrast, truncating variants in *MYH7*, *ACTN2,* and *PRDM16* were uniquely associated with LVNC and may reflect a distinct LVNC etiology. In particular, *MYH7* truncating variants (*MYH7*tv), generally considered nonpathogenic for cardiomyopathies, were 20-fold enriched in LVNC cases over controls. *MYH7*tv heterozygotes identified in the UK Biobank and healthy volunteer cohorts also displayed significantly greater noncompaction compared with matched controls. *RYR2* exon deletions and *HCN4* transmembrane variants were also enriched in LVNC, supporting prior reports of association with arrhythmogenic LVNC phenotypes.

**Conclusion:**

LVNC is characterized by substantial genetic overlap with DCM/HCM but is also associated with distinct noncompaction and arrhythmia etiologies. These results will enable enhanced application of LVNC genetic testing and help to distinguish pathological from physiological noncompaction.

## INTRODUCTION

Left ventricular noncompaction (LVNC) is a cardiomyopathy where the left ventricular myocardial wall is characterized by a compacted epicardial layer and a noncompacted and hypertrabeculated endocardial layer. LVNC is typically diagnosed in the context of left ventricular dysfunction when the ratio of noncompacted to compacted layer (NC/C) is greater than 2–2.3. As LVNC can occur either in conjunction with other cardiac diseases or as an isolated phenotype, its true nature is a matter of debate and conjecture.^1–3^ This is reflected by the different classifications assigned to LVNC by the American Heart Association^4^ (primary genetic cardiomyopathy) and the European Society of Cardiology^5^ (unclassified cardiomyopathy).

Notably, however, left ventricular hypertrabeculation can also occur in nonpathologic settings such as pregnancy or intensive athletic exercise^6,7^ where left ventricular function may be largely unaffected. Recent reports also indicate that up to 15% of individuals could meet NC/C ratio diagnostic criteria through cardiac magnetic resonance (CMR) imaging,^8^ highlighting the danger of overdiagnosis based on imaging alone.

LVNC is observed in patients with an array of genetic cardiac conditions, including cardiomyopathies, arrhythmias, aortopathies, and congenital heart disease,^9^ suggesting that it may represent a specific phenotypic trait in the presence of an underlying pathology rather than a distinct genetic cardiomyopathy. Initial reports of genetic variants identified in LVNC patients supported this theory, as most were in sarcomeric genes associated with hypertrophic cardiomyopathy (HCM) and dilated cardiomyopathy (DCM).^10^ The clinical surveillance of relatives of LVNC patients often detected features typical of cardiomyopathies other than LVNC,^11^ suggesting that other genetic or environmental factors often interact with a cardiomyopathy-predisposing variant to produce a noncompaction phenotype. However, the true nature and genetic etiology of LVNC, and whether it can be considered a separate disease entity, remains uncertain.

Several recent studies have used large panels of genes associated with inherited cardiac conditions to evaluate the genetic basis of LVNC.^12–15^ However, while providing valuable insights, these studies were individually underpowered to establish statistically robust single-gene associations, particularly for rarely causative genes. Additionally, the use of large panels that include genes not validated as causative in LVNC increases the risk of false positive associations, particularly where uniform variant classification criteria are applied for all genes. Recent re-evaluation of gene–disease associations for cardiomyopathies and other genetic diseases through initiatives such as ClinGen^16,17^ has refuted many earlier candidate gene studies, highlighting the need for more stringent methods to define disease genes.

We have recently used rare variant burden analysis to clarify the genetic basis of cardiomyopathies,^18,19^ and confirmed that genes characterized by a significant excess of rare variants in cases versus controls account for the vast majority of HCM patients with an identified pathogenic variant.^20^ These studies also demonstrated that genetic pleiotropy among cardiomyopathies had been overestimated and that largely distinct variant classes are associated with HCM (*MYBPC3*, *MYL2*, *MYL3*, *CSRP3*, *JPH2*, *FHOD3*, and nontruncating *FLNC*) and DCM (*TTN*, *LMNA*, *BAG3*, *RBM20*, *DSP*, *NEXN*, *VCL*, and truncating *FLNC*), with variants in *MYH7*, *TNNT2*, *TNNI3*, *TNNC1*, *TPM1*, *ACTC1*, and *PLN* robustly associated with both conditions.

Here we perform a meta-analysis of four published and two unpublished cohorts of sequenced LVNC cases to identify the genes and variant classes significantly associated with this condition. Such genes are likely to account for the preponderance of disease-causing variants in LVNC patients and should be prioritized for genetic testing. By comparing these findings with equivalent data from HCM/DCM cohorts, we can determine the extent to which LVNC is a distinct disease or a phenotypic expression of cardiomyopathies. We demonstrate substantial overlap between LVNC and DCM/HCM but also identify variant classes that are distinctly associated with LVNC and LVNC/arrhythmia phenotypes.

## MATERIALS AND METHODS

### LVNC cohorts

Six distinct cohorts were assessed in this study comprising 840 patients diagnosed with LVNC according to standard criteria who were referred for cardiac genetic testing (Table 1^10,12–14,21,22^). Two of these cohorts, Careggi University Hospital, Florence, Italy (*N* = 32) and the Laboratory for Molecular Medicine (LMM), Partners Healthcare, Boston, USA (*N* = 233), were previously unpublished. Four previously published cohorts were included: 327 probands from four Dutch cardiogenetic centers (van Waning et al.^13^), 95 probands from 13 French centers (Richard et al.^14^), 90 probands from a Polish/US study (Miszalski-Jamka et al.^12^), and 63 probands from a Swiss/German study (Klaassen et al.^10,21,22^). For previously published studies, we included only those where all rare variants detected in cases were listed, regardless of their diagnostic classification, to ensure accurate assessment of rare variant frequencies. See Supplemental Methods for additional details of each cohort and Table S1 for the number of cases sequenced per gene in each cohort.

**Table 1:** Details of the cohorts assessed in this study, including population/country of origin, number of LVNC probands, age profiles, number of genes sequenced/analyzed, and diagnostic inclusion criteria.

Study/center	Year	Population	Cases	Ages	Genes	Inclusion criteria
Careggi Hospital, Florence	–	Italy	32	–	81	Patients with a diagnosis of LVNC cardiomyopathy referred to the cardiac genetics service at Careggi University Hospital.
LMM, Boston	–	USA	233	95 ≥ 18 years 138 < 18 years	64	Patients referred for clinical genetic testing and a diagnosis of LVNC, excluding patients with indications that suggest a syndromic form of LVNC (i.e., including noncardiac symptoms) or only with *suspected* LVNC.
van Waning et al.^13^	2018	Netherlands	327	275 ≥ 18 years 52 < 18 years	66	LVNC cases referred to 4 cardiogenetic centers in Netherlands. Diagnosis based on consensus (by study author and another cardiologist) of re-evaluated echocardiography and MRI, according to the Jenni and Petersen criteria.
Richard et al.^14^	2019	France	95	–	107	Recent (≤6 months) diagnosis of isolated LVNC with echocardiography—multiple trabeculations with deep endomyocardial recesses, color Doppler evidence of perfused intertrabecular recesses, systolic NC/C >2, and no associated heart disease. Diagnosis reviewed by a core lab.
Miszalski-Jamka et al.^12^	2017	Poland/USA	90	–	104	Patients enrolled with known/suspected LVNC based on clinical presentation (history, symptoms, ECG, familial occurrence of LVNC) and 2-layered NC/C left ventricular myocardium by echocardiography. NC/C ratio >2.3 with cardiac MRI required for inclusion in this study.
Klaassen et al.^10,21,22^	2008 2011 2013	Swiss/German	63	–	9	LVNC patients referred to two tertiary centers. Diagnosis based on a NC/C ratio >2, prominent and excessive trabeculations, and deep intertrabecular recesses with perfusion by color Doppler imaging, in the absence of congenital heart anomalies.

See Supplemental Methods for additional details.

*ECG* electrocardiogram, *LMM* Laboratory for Molecular Medicine, *LVNC* left ventricular noncompaction, *MRI* magnetic resonance image, *NC/C* noncompacted to compacted layer.

### Rare variant burden testing for LVNC cases

Rare variant burden testing between case cohorts and gnomAD (exomes v2.1) population individuals (*n* = 125,748) was performed as previously described.^18^ Rare variants were defined as having a filtering allele frequency (FAF) in gnomAD <0.0001 (see Supplemental Methods for details). Analyses were performed separately for predicted truncating variants (nonsense, frameshift, and splice donor/acceptor variants) and nontruncating variants (missense, small inframe insertions/deletions, and stop lost). The frequency of rare variants in 70 genes (i.e., those sequenced and reported in at least half of the constituent disease cohorts to focus on the most relevant genes for LVNC) was compared between LVNC cases and gnomAD. All rare variants detected in LVNC cases were included in burden testing regardless of the clinical classification applied in any of the constituent cohorts. The number of LVNC cases sequenced per gene ranged from 173 to 820. For gnomAD, the denominator was adjusted for each gene to account for variable coverage in exome-sequenced samples, as described in Supplemental Methods. Statistical significance for enrichment of variants in cases was assessed with a one-sided Fisher’s exact test, with Bonferroni correction applied for testing 70 genes. The case excess was defined as the difference in rare variant frequencies between case cohorts and gnomAD. The full list of rare variants detected in each LVNC cohort is provided in Table S2.

For genes not enriched for rare variants in LVNC versus gnomAD, we tested for potential domain-specific enrichment of nontruncating variants using an unsupervised, sequence-based clustering algorithm^23^ (details in Table S3). Furthermore, we also assessed variant enrichment in cases for the established *RBM20* DCM pathogenic hotspot (residues 634–638). In addition, we analyzed the occurrence of structural variants (SVs) in *RYR2* in LVNC cases and controls, based on previously published reports (further details below and in Supplemental Methods).

### Comparison with gene associations in other cardiomyopathies

Variant classes with a significant excess in LVNC versus gnomAD were compared with the results of similar analyses in DCM and HCM cohorts^18,20^ (see Supplemental Methods and Table S4 for details of the cohorts used). Variant classes enriched in LVNC patients as well as in DCM and/or HCM indicate a potential shared genetic etiology between LVNC and DCM/HCM, whereas those unique to LVNC suggest a distinct etiology.

### Effect of *MYH7* truncating variants in population controls

The CMR-derived maximum NC/C ratios in individuals with a *MYH7* truncating variant (*MYH7*tv) in the UK Biobank (*n* = 12,447 individuals with both exome sequencing and CMR imaging) and healthy volunteers from the UK Digital Heart Project (*n* = 912)^24^ and the Egyptian Collaborative Cardiac Genomics (ECCO-GEN) Project (*n* = 400)^25^ were compared with an equivalent number of year of birth–, sex-, and ethnicity-matched *MYH7*tv-negative individuals to assess the effect of these variants on noncompaction (Figure S1 and further details in Supplemental Methods). Significance was assessed with a one-sided Wilcoxon rank-sum test.

## RESULTS

### Rare variant burden in LVNC cases versus gnomAD

**Fig. 1:**
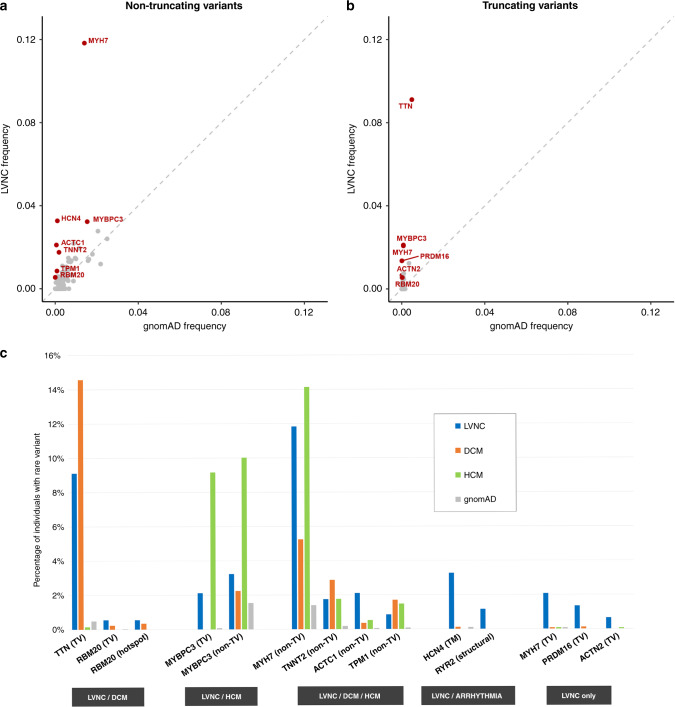
Significant genetic associations for LVNC and other cardiomyopathies. (**a**,**b**) Comparison of the frequency of rare variants for the combined left ventricular noncompaction (LVNC) cohorts (*y*-axis) and gnomAD individuals (*x*-axis) for nontruncating variants (**a**) and truncating variants (**b**). Genes with a significant excess in LVNC (*p* < 0.0007 with Bonferroni correction) are highlighted in red. For nontruncating variants, data are restricted to the transmembrane region and pathogenic hotspot for *HCN4* and *RBM20* respectively, as described in the text. (**c**) Variant classes with a significant excess in LVNC cases versus gnomAD, with comparison to equivalent frequencies in dilated cardiomyopathy (DCM) and hypertrophic cardiomyopathy (HCM) cohorts. Variant classes are grouped according to which conditions display significant enrichment over gnomAD—LVNC and DCM, LVNC and HCM, all three indications, LVNC and arrhythmia phenotypes and LVNC only. Variant classes shown are truncating variants (TV), nontruncating variants (non-TV) and pathogenic hotspot for *RBM20*, transmembrane region (TM) for *HCN4* and structural variants for *RYR2*.

To investigate the genes and variant classes associated with LVNC, we compared the frequency of rare variation in six LVNC cohorts with gnomAD exomes population controls. A significant excess of rare variants in LVNC cases compared with gnomAD (*p* < 0.0007 with Bonferroni adjustment for testing 70 genes) was observed for truncating variants in *TTN* (excess burden in cases = 8.6%), *MYBPC3* (2.0%), *MYH7* (2.0%), *PRDM16* (1.4%), *ACTN2* (0.6%), and *RBM20* (0.5%), for nontruncating variants in *MYH7* (10.4%), *ACTC1* (2.0%), *MYBPC3* (1.7%), *TNNT2* (1.6%), *TPM1* (0.8%), and structural variants (SVs) in *RYR2* (1.2%, all exon deletions) (Fig. 1 and Table 2,^26^ for full details on all genes see Table S5). Although nontruncating variants were not significantly enriched overall for *RBM20* and *HCN4*, a significant excess was observed for the DCM pathogenic hotspot in *RBM20* (0.5%) and the transmembrane region of *HCN4* (3.2%). Based on the cumulative excess of these significantly enriched variant classes, a causative genetic variant would be identified in an estimated 36.6% of LVNC cases, in line with contemporary estimates for other cardiomyopathies.^18,19^

**Table 2:** Variant classes with a significant excess (after Bonferroni multiple testing correction) of rare variants in combined LVNC cohorts compared with gnomAD exomes.

Gene	Variant class	Other cardiac associations	gnomAD count/total	gnomAD frequency	LVNC count/total	LVNC frequency	Case excess	*p* value (nominal)	Odds ratio (95% CI)
*MYH7*	Nontruncating	DCM, HCM	1,754/124,979	1.40%	97/820	11.83%	10.43%	7.7E-56	9.4 (7.6-11.7)
*TTN*	Truncating	DCM	590/122,054	0.48%	47/516	9.11%	8.63%	1.2E-42	20.6 (15.1–28.1)
*ACTC1*	Nontruncating	DCM, HCM	73/125,274	0.06%	16/760	2.11%	2.05%	3.1E-19	36.9 (21.4–63.7)
*MYBPC3*	Truncating	HCM	89/115,675	0.08%	17/805	2.11%	2.04%	1.7E-18	28.0 (16.6–47.3)
*MYH7*	Truncating	–	97/124,979	0.08%	17/820	2.07%	1.99%	2.4E-18	27.3 (16.2–45.8)
*PRDM16*	Truncating	–	7/120,147	0.01%	6/444	1.35%	1.35%	4.0E-12	235.1 (78.7–702.4)
*TNNT2*	Nontruncating	DCM, HCM	227/124,805	0.18%	13/741	1.75%	1.57%	2.8E-09	9.8 (5.6–17.2)
*HCN4* (tm)	Nontruncating	Bradycardia	107/103,942	0.10%	7/214	3.27%	3.17%	4.8E-09	32.8 (15.1–71.4)
*RYR2*	Exon deletions	CPVT	0/10,738	0.00%	5/429	1.17%	1.17%	8.2E-08	278.3 (15.4–5040.7)
*ACTN2*	Truncating	–	13/125,085	0.01%	4/611	0.66%	0.65%	1.3E-06	63.4 (20.6–195.0)
*RBM20* (hs)	Nontruncating	DCM	1/76,260	0.00%	3/546	0.55%	0.55%	1.4E-06	421.3 (43.8–4056.9)
*TPM1*	Nontruncating	DCM, HCM	105/124,430	0.08%	6/702	0.85%	0.77%	4.2E-05	10.2 (4.5–23.3)
*RBM20*	Truncating	DCM	18/76,260	0.02%	3/546	0.55%	0.53%	4.3E-04	23.4 (6.9–79.7)
*MYBPC3*	Nontruncating	HCM	1,780/115,675	1.54%	26/805	3.23%	1.69%	4.6E-04	2.1 (1.4–3.2)

For *TTN*, only variants affecting exons included in >90% of the transcripts (percent spliced in [PSI] >0.9) were included.^26^ For *HCN4* and *RBM20*, only variants within the transmembrane region and pathogenic hotspot respectively were included, as described in “Materials and Methods.” For *RYR2*, only structural variants (exon deletions) are noted and compared with equivalent variants in gnomAD genomes. Reported *p* values are nominal, with the Bonferroni-corrected significance threshold is 0.05/70 (7.1E-04).

*CI* confidence interval, *CPVT* catecholaminergic polymorphic ventricular tachycardia, *DCM* dilated cardiomyopathy, *HCM* hypertrophic cardiomyopathy, *LVNC* left ventricular noncompaction.

Three variant classes are nominally associated with age of onset: *TTN* truncating (*p* = 0.013) and *ACTC1* nontruncating (*p* = 0.008) variants are enriched in adults and children respectively (consistent with prior reports for DCM^19,27^), while there is a trend for enrichment of *MYH7*tv in pediatric cases (*p* = 0.053) (Supplemental Methods, Table S6).

### Overlap with variant classes associated with HCM and DCM

**Fig. 2:**
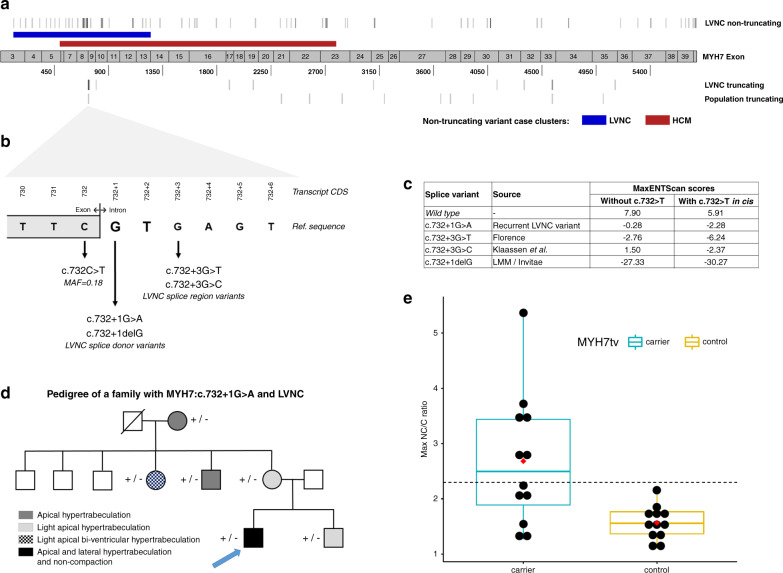
Positional, molecular, and clinical characterization of *MYH7* truncating variants (*MYH7*tv) in left ventricular noncompaction (LVNC) and in the population. (**a**) Distribution of *MYH7* nontruncating variants demonstrates distinct (though overlapping) enriched clusters in LVNC (blue band) and hypertrophic cardiomyopathy (HCM)^23^ (red band). *MYH7*tv are distributed throughout the transcript in LVNC and population cohorts but with a cluster around the c.732 splice region. (**b**) Details of the c.732 splice region and associated variants found in LVNC cases. (**c**) MaxEntScan^29^ scores for these variants (and the wild type sequence) with the reference exon base at c.732 and the c.732C>T common variant in *cis*. (**d**) Pedigree of the Italian family demonstrated segregation of the c.732+1G>A variant with noncompaction and/or varying degrees of myocardial hypertrabeculation. (**e**) *MYH7*tv are associated with higher noncompacted to compacted (NC/C) ratios in population cohorts. Maximum NC/C ratios of individuals identified with *MYH7*tv in population controls not selected for disease (see “Materials and Methods”), compared with age- and sex-matched individuals without MYH7tv drawn from the same populations. Boxplots show the median and interquartile range, red diamond indicates mean and the dashed line shows the diagnostic NC/C ratio of 2.3.

The variant classes enriched in LVNC were compared with those enriched in DCM and HCM (Fig. 1c), to enable assessment of the genetic overlap with LVNC. Truncating variants in *TTN* and *RBM20*, as well as nontruncating variants within the pathogenic DCM hotspot of *RBM20*, are significantly enriched in both LVNC and DCM. Truncating and nontruncating variants in *MYBPC3* are enriched in both LVNC and HCM. The proportion of LVNC cases with variants in *TTN* and *MYBPC3* is notably lower compared with DCM and HCM, respectively, which may reflect the more heterogeneous etiology of LVNC. A significant excess of nontruncating variants in four other sarcomeric genes (*MYH7*, *TNNT2*, *TPM1*, *ACTC1*) is observed in all three conditions. Of the enriched variant classes, nontruncating variants in *MYH7* (also commonly observed in DCM and HCM) had the highest frequency in LVNC cases (11.8%). However, distinctive (though overlapping) patterns of variant clustering were observed in LVNC and HCM cohorts (Fig. 2a), with variants in LVNC cases clustered around the N-terminus myosin head region (residues 39–415).

### Variant classes unique to LVNC

Five variant classes were found to be significantly enriched solely in LVNC cases, indicating such variants may yield a distinct noncompaction phenotype unrelated to either DCM or HCM - truncating variants in three genes (*MYH7*, *PRDM16*, and *ACTN2*) and specific variant classes in two arrhythmia-associated genes (*RYR2* and *HCN4*).

### *MYH7* truncating variants

*MYH7*tv occur in 2.1% of LVNC cases compared with 0.08% in gnomAD (*p* = 2.4E-18) and are observed in each of the six LVNC cohorts (Table 3,^28^ Table S7). These include a single splice donor variant, c.732+1G>A, present in six cases (with three other variants at this splice junction), as well as ten other nonsense, frameshift, and splice acceptor variants. Significant enrichment of *MYH7*tv remains when excluding the c.732 splice junction variants (*p* = 2.4E-09), which, along with the variant distribution throughout the *MYH7* transcript (Fig. 2a), suggests that such variants are generally pathogenic in LVNC with haploinsufficiency a likely mechanism of action.

**Table 3:** Details of *MYH7* truncating variants detected in LVNC cases in the six cohorts used in this meta-analysis.

CDS	Protein	Population/cohort	Cases	Clinical and family details
**c.732 splice region**				
c.732+1G>A	–	Netherlands	2	Variant detected in father, 10-year-old son, and newborn with LVNC, absent in 2 unaffected sisters of father.^28^ Family of Turkish origin.
		Switzerland/Germany	2	Family LVNC-101: detected in 6 affected, absent in 8 unaffected, LOD = 2.6. Family LVNC-108: detected in 3 affected, absent in 1 unaffected. Variants reported as c.818+1G>A. Haplotype analysis suggested the variants arose independently.
		Italy	1	Variant detected in 6 family members with varying degrees of noncompaction and/or hypertrabeculation (Fig. 2d). None had the c.732C>T common variant.
		USA/LMM	1	Male, 0 months, cystic hygroma, Ebstein anomaly, LVNC, and family history of LVNC. Variant also detected in twin brother with LVNC. Both had the c.732C>T common variant in *trans*. Both parents are unaffected. Unspecified ethnicity.
c.732+1delG	–	USA/LMM	1	Female, 0 months, clinical diagnosis of LVNC with severely dilated left atrium and small secundum ASD, paternal family history of “enlarged heart” (father not tested) maternal family history of “fainting episodes” though variant not detected in mother.
c.732+3G>T	–	Italy	1	Detected in the affected mother (c.732C>T not detected) of an affected 5-year-old son.
c.732+3G>C	–	Switzerland/Germany	1	Family LVNC-109: detected in 2 affected, absent in 1 unaffected. Reported as c.818+3G>C.
**Other** ***MYH7*** **truncating variants**				
c.745C>T	p.Arg249X	Poland/USA	1	–
c.798T>A	p.Tyr266X	Netherlands	1	Detected in girl, 4 years, and affected father (grandfather died suddenly at 60).^28^
c.1903A>T	p.Lys635X	Poland/USA	1	–
c.2085_2097dup	p.Glu700Glnfs*37	Netherlands	1	–
c.3100-2A>C	–	Netherlands	1	–
c.4125T>A	p.Tyr1375X	Netherlands	1	–
c.4354-2A>C	–	USA/LMM	1	Male, 15 years, clinical diagnosis of LVNC with biventricular dilation and PVCs.
c.4588C>T	p.Arg1530X	France	1	–
		USA/LMM	1	Female, 15 years, clinical diagnosis of LVNC.
c.5110C>T	p.Gln1704X	USA/LMM	1	Male, 0 months, with Ebstein anomaly and LVNC, family history of CHD (septal defect) and arrhythmia. Variant in *trans* with *MYH7*:p.Arg1897His. Both parents unaffected.

*ASD* atrial septal defect, *CHD* congenital heart disease, *LMM* Laboratory for Molecular Medicine, *LOD* logarithm of the odds, *LVNC* left ventricular noncompaction, *PVC* premature ventricular contractions.

The c.732+1G>A splice donor variant was detected in six individuals of different nationalities/ethnicities, indicating it is not a founder variant but one that has occurred recurrently in several different families and populations (Table 3). It is significantly enriched in LVNC cases compared with gnomAD (1/125,745 individuals in gnomAD exomes v2.1, *p* = 5.1E-13). To investigate this variant further we examined the pedigree of the Italian patient in more detail. The proband is a 31-year-old male who was diagnosed with LVNC at 14 years of age. Positive family history for LVNC was reported in the mother’s family. Targeted sequencing, echocardiography, and electrocardiogram (ECG) were performed on the proband, his brother, and four maternal family members. The variant was detected in all six family members, all showed varying degrees of myocardial hypertrabeculation (Fig. 2d). We attempted to assess the effect of this variant on *MYH7* transcription using RNA from blood lymphocytes (other tissue was not available) but were unable to amplify any product (Supplemental Methods).

Three other variants in this splice region were detected in LVNC cases (Table 3, Fig. 2b), all of which are predicted to affect splicing by the MaxEntScan algorithm^29^ (Fig. 2c). No other variants in this splice region (intronic +1 to +8 bases) are detected in gnomAD exomes (v2.1) or genomes (v3). Interestingly, the adjacent synonymous exonic splice region variant, c.732C>T, is the only common splice region variant in *MYH7* (minor allele frequency [MAF] = 0.18) and is predicted by MaxEntScan to further disrupt splicing if co-occurring with the rare splice variants (Fig. 2c). Whether there is a connection between the recurrence of rare splice variants in this splice region in LVNC cases and the presence of a common variant at the exon–intron boundary will require further investigation. However, no enrichment of the common splice region variant (c.732C>T) was observed in LVNC compared with ethnicity-matched gnomAD individuals (Table S8).

We then assessed NC/C ratios of individuals from the UK Biobank and healthy volunteer cohorts with *MYH7*tv. Of 12 heterozygotes, 6 had ratios >2.3 (the diagnostic criteria for LVNC). The NC/C ratio was significantly greater in heterozygotes compared with matched *MYH7*tv-negative individuals in these cohorts (2.7 ± 1.2 vs. 1.6 ± 0.3, *p* = 0.0034) (Fig. 2e, Table S9).

### *PRDM16* and *ACTN2* truncating variants

The truncating variants in *PRDM16* (occurring in 1.4% of LVNC cases) include two variants previously published in the Swiss/German cohort,^22^ three variants in the Dutch cohort,^13^ and one variant in the LMM cohort (Table S10). Three truncating variants in *ACTN2* were observed in the Dutch cohort and one variant, p.Arg192X, in the Italian cohort. These findings add to existing evidence (Table S11 and “Discussion”) for a role for *PRDM16* and *ACTN2* in LVNC. Notably, both genes are defined as loss-of-function intolerant (pLI = 1) in gnomAD, with fewer observed than expected variants for *PRDM16* (o/e ratio=0.08) and *ACTN2* (0.12), offering additional supportive evidence for the deleteriousness of these variant classes.

### Variants in arrhythmia-associated genes: *RYR2* and *HCN4*

Variants in *RYR2* are the primary cause of catecholaminergic polymorphic ventricular tachycardia (CPVT), with pathogenic missense variants present in approximately 50% of cases. Deletion of exon 3 in *RYR2* has been described in CPVT^30^ and recently in a number of patients and families with complex phenotypes that include LVNC and CPVT (Table S11). In this meta-analysis, five *RYR2* exon deletions were observed in LVNC cases (three of exon 3 and one each of exons 2 and 19) (Table S10). No *RYR2* exon deletions were detected in 14,891 gnomAD genome-sequenced individuals,^31^ suggesting such variants are rarely observed in the population.

The overall enrichment of nontruncating variants in *HCN4* (3.7% vs 1.1%) did not meet the significance threshold with Bonferroni correction (*p* = 0.002). However, we found significant clustering of nontruncating variants in the transmembrane region of the ion channel encoded by *HCN4* (*p* = 3.5E-07, Table S3) and therefore performed burden testing for variants within and outside this region. While no enrichment occurred outside the transmembrane region (*p* = 0.87), a significant excess was observed for variants within this region (3.3% vs. 0.1%, *p* = 4.8E-09). Bradycardia was frequently observed in LVNC cases with *HCN4* transmembrane variants, including 3/5 probands in the French study (Table S10). In contrast, the Italian LVNC patient with the only nontransmembrane *HCN4* variant detected in this study (p.Gly1077Ser) had a normal ECG, suggesting this variant is unlikely to be disease-causing.

### Variant interpretation for LVNC

Of 208 distinct rare variants in the enriched LVNC variant classes, 62 are classified as (likely) pathogenic for LVNC, HCM, or DCM (ClinVar version 201909) (Table S2). Recommendations for LVNC-specific adaptions of variant interpretation guidelines are described in Table S12.

## DISCUSSION

The meta-analysis of genetic sequencing data from 840 cases described here provides much needed clarity concerning the genetic basis of LVNC. By amalgamating data from several recently published and moderately sized studies with two new cohorts, we were able to identify genes and variant classes with robust statistical evidence of association with LVNC. These findings highlight the diverse etiology underlying this phenotype and inform how genetic testing should be applied and interpreted for patients presenting with LVNC.

Our results reveal a substantial overlap in genes and variant classes enriched in LVNC with those of the more genetically well-defined cardiomyopathies of DCM and HCM. These findings are consistent with the increasingly held view that LVNC largely belongs to the spectrum of more established cardiomyopathies. The expression of the particular trait (i.e., hypertrabeculation) represents a striking phenotypic variation whose impact on the pathophysiology and natural history of the underlying paradigm (HCM or DCM) remains unclear, although recent management consensus documents recommend assessing risk and treating LVNC according to the principles of HCM or DCM, as appropriate.

The factors that cause patients with DCM/HCM-causing variants to develop and/or present with LVNC are unknown but could involve genetic and nongenetic modifiers. One intriguing candidate is the *MIB1* gene, a regulator of the Notch signaling pathway that has previously been implicated in LVNC.^32^ Three *MIB1* truncating variants were identified in the Dutch cohort and notably all co-occur with *TTN* truncating variants, suggesting they could modify the DCM phenotype typically associated with *TTN* variants.^13^ The relatively high frequency of *MIB1* truncating variants in the population (*MIB1* has the tenth lowest pLI score of all human genes in gnomAD and an o/e ratio of 1.83) support the hypothesis that they may act as modifiers rather than primary pathogenic variants.

Detailed genotype–phenotype studies of LVNC cases and their family members will be required to fully clarify the extent of this phenotypic overlap between cardiomyopathies. However, a recent study assessing clinical and genetic screening in families of LVNC patients from the Dutch cohort provided further evidence for this hypothesis.^33^ Many family members had DCM or HCM without noncompaction, and the genotype of the proband was broadly predictive of the phenotype in relatives*—TTN* and *MYH7* tail domain variants were associated with DCM in relatives and *MYBPC3* variants were associated with HCM. In contrast, the authors found that *MYH7* head domain variants in probands were predictive of isolated LVNC in relatives, indicating that specific variant classes may be associated with a distinctive noncompaction phenotype rather than underlying DCM/HCM.

Accordingly, we detected additional variant classes not associated with other cardiomyopathies but enriched in LVNC patients, potentially explaining 5–10% of cases. This patient subset may therefore have an etiology separate from other cardiac conditions and represent genetically distinct disease where noncompaction is the primary or presenting phenotype.

Of the variant classes unique to LVNC, perhaps the most notable are truncating variants in *MYH7*. Such variants have generally been considered nonpathogenic and indeed are not associated with either HCM or DCM,^18^ where nontruncating (largely missense) variants act through a dominant negative mechanism (with opposing activating and inactivating functions). However, they are observed in >2% of LVNC cases, consistently across all of the cohorts analyzed here, and are significantly enriched over the gnomAD population rate (*p* = 2.4E-18). Data from population cohorts provided further supporting evidence for the role of *MYH7*tv in noncompaction, with NC/C ratios significantly greater in *MYH7*tv heterozygotes compared with matched controls and 50% meeting the NC/C diagnostic criteria for LVNC, suggesting a relatively high population-level penetrance. The exact mechanism of action of these variants remains to be determined although their distribution throughout the *MYH7* gene would support nonsense-mediated decay and haploinsufficiency. The large number of variants clustering around one splice region is particularly intriguing as the most common of these, c.732+1G>A, does not appear to be a founder variant. More research is required to establish why variants in this location are particularly associated with LVNC.

Truncating variants in two other genes, *ACTN2* and *PRDM16*, also appear to be associated primarily with an LVNC phenotype. These observations are supported by other associations with LVNC at these loci (Table S11), e.g., the exon 3–6 deletion in *ACTN2* detected in an LVNC patient and the LVNC phenotype underlying 1p36 deletion syndrome that may involve *PRDM16*.^22,34^ Although no excess of *ACTN2* rare variants (truncating or nontruncating) have been observed in DCM or HCM cohorts,^18,20^ two missense variants have been reported in pedigrees with complex heterogeneous phenotypes that include noncompaction (Table S11). The significant association of *ACTN2* truncating variants with LVNC described here may indicate that such loss-of-function variants in this gene lead to more overt presentation of LVNC.

This study has shown that prior reports of *RYR2* and *HCN4* variants with LVNC and CPVT or bradycardia, respectively, are supported by statistically significant associations in case–control cohort analysis, albeit when assessing specific variant classes. The pathogenicity of the *RYR2* exon 3 deletion has been established in several reports (Table S11), but the deletions of two other *RYR2* exons (2 and 19) described here suggest a potentially broader role for this variant class in LVNC (although the pathogenicity of these novel variants remains to be unambiguously established). The enrichment of missense variants in the *HCN4* transmembrane region is also consistent with previous reports describing combined LVNC/arrhythmia phenotypes (Table S11). The reports on both of these variant classes reveal considerable phenotypic heterogeneity but it is conceivable that such patients could present primarily with an LVNC phenotype.

Despite the significant gene–disease associations described here, it should be noted that no significant excess of rare variation was observed for the majority of the analyzed genes, similar to previous findings for HCM^20^ and DCM.^18^ While a lack of excess does not necessarily preclude a role in disease, it does indicate that variants in such genes are likely to be, at best, very rarely causative.

The findings of this study enable evidence-based design of LVNC genetic testing panels that accounts for its diverse etiology but restricts testing to those genes with a proven association with disease to minimize uncertainty and false positive results. Our results suggest it may be prudent to include all valid DCM/HCM genes in LVNC genetic testing (whether validated though statistical association^18,20^ or evidence curation^17,20^), including established DCM/HCM genes with only nominal enrichment in LVNC cases (Table S5). Our results will also inform interpretation of genetic testing results for LVNC cases, helping to identify the underlying etiology (DCM, HCM, or isolated LVNC) and informing clinical management for patients and their families. For example, detecting pathogenic *RYR2* or *HCN4* variants in patients presenting with LVNC could identify those cases (and their family members) at risk of potentially severe arrhythmogenic events. More accurate detection of pathogenic LVNC variants may also help to distinguish between pathological and physiological noncompaction, an increasingly important task given the potential for overdiagnosis based on imaging diagnostic criteria alone.

### Limitations

There are some limitations associated with the analysis described here. As this is a meta-analysis of six different LVNC cohorts, there may be minor differences in how LVNC was diagnosed between the different studies and in the inclusion or exclusion criteria for patients with other cardiac phenotypes in addition to LVNC. However, we observe broad consistency across cohorts for the significantly associated variant classes (e.g., *MYH7*tv, Table S7), despite the limited cohort sizes, indicating this is not a major confounding factor. Burden testing was performed comparing data from different platforms, which may introduce bias when comparing cases with reference population samples. As described previously, we adjusted for expected poorer coverage in the exome sequencing data of gnomAD^18^ and used FAF values in gnomAD to define rarity, so to minimize any confounding effects due to population stratification. Our previous work for HCM showing strong correlation between genes validated through this approach^18,20^ and those validated by the curation of published evidence^17,20^ demonstrates its robustness for identifying the most relevant causative genes in Mendelian diseases. Future larger single-center studies or coordinated efforts between different centers that synchronize diagnostic criteria and sequencing methods will be valuable in confirming the gene associations described here and clarifying any role for other genes and variant classes in LVNC.

### Conclusions

By identifying significant genetic associations with LVNC, we are able to clarify the nature of this complex and enigmatic phenotype. These findings confirm a large genetic overlap with other cardiac conditions, supporting the hypothesis that many LVNC cases are a variable morphological phenotype of an underlying cardiac disease, and also identify a distinct genetic etiology in a subset of cases. Our results indicate that focused genetic testing in patients that present with LVNC may distinguish between different etiologies and guide clinical management for patients and their relatives. This study also demonstrates the power of statistically robust genetic association studies in characterizing complex clinical phenotypes.

## Supplementary information

Supplemental Methods

Supplementary table 1

## Data Availability

The data are not deposited in public repositories but all data are available in Supplemental data.

## References

[1] Anderson RH (2017). Key questions relating to left ventricular noncompaction cardiomyopathy: is the emperor still wearing any clothes?. Can. J. Cardiol..

[2] Oechslin E, Jenni R (2017). Nosology of noncompaction cardiomyopathy: the emperor still wears clothes!. Can. J. Cardiol..

[3] Hershberger, R. E., Morales, A. & Cowan, J. Is left ventricular noncompaction a trait, phenotype, or disease? The evidence points to phenotype. *Circ. Cardiovasc. Genet.* **10**, e001968 (2017). 10.1161/CIRCGENETICS.117.001968 29212902

[4] Maron BJ (2006). Contemporary definitions and classification of the cardiomyopathies: an American Heart Association Scientific Statement from the Council on Clinical Cardiology, Heart Failure and Transplantation Committee; Quality of Care and Outcomes Research and Functio. Circulation..

[5] Elliott P (2008). Classification of the cardiomyopathies: a position statement from the European Society Of Cardiology Working Group on Myocardial and Pericardial Diseases. Eur. Heart J..

[6] Gati S (2014). Reversible de novo left ventricular trabeculations in pregnant women: implications for the diagnosis of left ventricular noncompaction in low-risk populations. Circulation..

[7] Gati S (2013). Increased left ventricular trabeculation in highly trained athletes: do we need more stringent criteria for the diagnosis of left ventricular noncompaction in athletes?. Heart..

[8] Ross SB (2020). A systematic review and meta-analysis of the prevalence of left ventricular noncompaction in adults. Eur. Heart J..

[9] Biagini E (2006). Different types of cardiomyopathy associated with isolated ventricular noncompaction. Am. J. Cardiol..

[10] Klaassen S (2008). Mutations in sarcomere protein genes in left ventricular noncompaction. Circulation..

[11] Murphy RT (2005). Natural history and familial characteristics of isolated left ventricular noncompaction. Eur. Heart J..

[12] Miszalski-Jamka, K. et al. Novel genetic triggers and genotype-phenotype correlations in patients with left ventricular noncompaction. *Circ. Cardiovasc. Genet.* **10**, e001763 (2017). 10.1161/CIRCGENETICS.117.001763 PMC5665372 28798025

[13] van Waning JI (2018). Genetics, clinical features, and long-term outcome of noncompaction cardiomyopathy. J. Am. Coll. Cardiol..

[14] Richard P (2019). Targeted panel sequencing in adult patients with left ventricular noncompaction reveals a large genetic heterogeneity. Clin. Genet..

[15] Miller, E. M. et al. Genetic testing in pediatric left ventricular noncompaction. *Circ. Cardiovasc. Genet.* **10**, e001735 (2017). 10.1161/CIRCGENETICS.117.001735 PMC8428628 29212898

[16] Strande NT (2017). Evaluating the clinical validity of gene-disease associations: an evidence-based framework developed by the Clinical Genome Resource. Am. J. Hum. Genet..

[17] Ingles J (2019). Evaluating the clinical validity of hypertrophic cardiomyopathy. Genes Circ. Genomic Precis. Med..

[18] Walsh R (2017). Reassessment of Mendelian gene pathogenicity using 7,855 cardiomyopathy cases and 60,706 reference samples. Genet. Med..

[19] Mazzarotto F (2020). Reevaluating the genetic contribution of monogenic dilated cardiomyopathy. Circulation..

[20] Walsh R (2017). Defining the genetic architecture of hypertrophic cardiomyopathy: re-evaluating the role of non-sarcomeric genes. Eur. Heart J..

[21] Probst S (2011). Sarcomere gene mutations in isolated left ventricular noncompaction cardiomyopathy do not predict clinical phenotype. Circ. Cardiovasc. Genet..

[22] Arndt A-K (2013). Fine mapping of the 1p36 deletion syndrome identifies mutation of PRDM16 as a cause of cardiomyopathy. Am. J. Hum. Genet..

[23] Walsh, R. et al. Quantitative approaches to variant classification increase the yield and precision of genetic testing in Mendelian diseases: the case of hypertrophic cardiomyopathy. *Genome Med*. **11**, 5 (2019). 10.1186/s13073-019-0616-z PMC6350371 30696458

[24] de Marvao A (2015). Precursors of hypertensive heart phenotype develop in healthy adults. JACC Cardiovasc. Imaging..

[25] Aguib, Y. et al. The Egyptian Collaborative Cardiac Genomics (ECCO-GEN) Project: defining a healthy volunteer cohort. *NPJ Genom. Med*. **5**, 46 (2020). 10.1038/s41525-020-00153-w PMC7584615 33110626

[26] Roberts AM (2015). Integrated allelic, transcriptional, and phenomic dissection of the cardiac effects of titin truncations in health and disease. Sci. Transl. Med..

[27] Fatkin D (2016). Titin truncating mutations: a rare cause of dilated cardiomyopathy in the young. Prog. Pediatr. Cardiol..

[28] Hoedemaekers YM (2010). The importance of genetic counseling, DNA diagnostics, and cardiologic family screening in left ventricular noncompaction cardiomyopathy. Circ. Cardiovasc. Genet..

[29] Yeo G, Burge CB (2004). Maximum entropy modeling of short sequence motifs with applications to RNA splicing signals. J. Comput. Biol..

[30] Bhuiyan ZA (2007). Expanding spectrum of human RYR2-related disease: new electrocardiographic, structural, and genetic features. Circulation..

[31] Collins RL (2020). A structural variation reference for medical and population genetics. Nature.

[32] Luxán G (2013). Mutations in the NOTCH pathway regulator MIB1 cause left ventricular noncompaction cardiomyopathy. Nat. Med.

[33] van Waning, J. I., Caliskan, K., Michels, M. et al. Cardiac Phenotypes, Genetics, and Risks inFamilial Noncompaction Cardiomyopathy. *J Am Coll Cardiol*. **73** 1601–1611 (2019). 10.1016/j.jacc.2018.12.085 30947911

[34] Singer, E. S., Ross, S. B., Skinner, J. R. et al. Characterization of clinically relevant copy-numbervariants from exomes of patients with inherited heart disease and unexplained suddencardiac death. *Genet Med.* (2020). 10.1038/s41436-020-00970-5. 10.1038/s41436-020-00970-5 32973354

