# Supplementary material for: Systematic large-scale assessment of the genetic architecture of left ventricular noncompaction reveals diverse etiologies
Source: Genet Med. 2021 Jan 26;23(5):856–64. doi: 10.1038/s41436-020-01049-x (PMC8105165; doi:10.1038/s41436-020-01049-x)
Supplement: Supplementary file 1 — Supplemental Methods [file 41436_2020_1049_MOESM1_ESM.pdf]

Systematic large-scale assessment of the genetic architecture of left ventricular non-compaction reveals diverse aetiologies

Supplemental Methods

LVNC cohorts included in this study

Two of the six cohorts analysed were unpublished:

- 32 unrelated cases with a diagnosis of LVNC cardiomyopathy were sequenced by the genetics laboratory at Careggi University Hospital in Florence, Italy. All patients underwent genetic testing from 2013 onwards, with those tested until mid-2017 (N=27) sequenced on a panel comprising 111 genes implicated in cardiomyopathies, and those tested later (N=5) sequenced on the 174-genes Illumina TruSight Cardio panel (Table S1). All participants gave written informed consent and the study was approved by the Comitato Etico Area Vasta Centro (CEAVC).
- 233 unrelated cases referred for clinical genetic testing and with a definitive diagnosis of LVNC were sequenced by the Laboratory of Molecular Medicine, Partner's Healthcare, Boston, USA. Patients with indications that suggest a syndromic form of LVNC (i.e. including non-cardiac symptoms) or only with *suspected* LVNC were excluded from the analysis. As the gene panels used evolved over time, the number of cases sequenced ranged from 36 to 231 depending on the gene (Table S1). Data collection for this study was approved by the Partners HealthCare Institutional Review Board (protocol 2006P001108: Genotype-Phenotype Studies for Cardiomyopathies) and all data was de-identified prior to analysis.

The other four cohorts analysed were previously published:

- van Waning *et al.* described a cohort of up to 327 LVNC probands from four cardiogenetic centres in the Netherlands[1], sequenced on panels of cardiomyopathy genes. LVNC diagnosis was based on evaluation of echocardiography and magnetic resonance imaging using the Jenni and Petersen criteria. Up to 66 genes were sequenced in each case.
- Richard *et al.* described a cohort of 95 adult LVNC probands enrolled between 2012 and 2013 in 13 French centres for inherited cardiac diseases[2], sequenced on a 107 gene cardiomyopathy and arrhythmia panel. All patients had a recent diagnosis of isolated LVNC (within 6 months of inclusion in the study) with echocardiography reviewed by a core lab to confirm diagnosis.
- Miszalski-Jamka *et al.* described a cohort of 174 probands[3] – 90 were diagnosed with LVNC (a two-layered trabeculated and compacted LV myocardium with maximum NC/C ratio >2.3:1) with 84 cases described as having left ventricular hypertrabeculation (LVHT), i.e. cases that failed to meet the criteria for LVNC diagnosis but were suspected of having LVNC. Only the 90 definitive LVNC cases in this cohort were used for this meta-analysis. Exome sequencing was performed for each case but variant details for only 104 cardiac genes were published and included in this study.
- Sixty-three LVNC cases from Swiss and German centres were assessed by Klaassen *et al.*[4] (sequencing of 6 sarcomeric genes - *MYH7*, *TNNT2*, *TNNI3*, *ACTC1*, *MYL2*, *MYL3*) and Probst *et al*[5] (sequencing of 2 sarcomeric genes – *MYBPC3*, *TPM1*). These cases were diagnosed with established criteria, a NC/C ratio >2 with prominent trabeculations and deep intertrabecular recesses, in the absence of congenital heart anomalies. An expanded version of this cohort, comprising 75 LVNC cases, were sequenced for *PRDM16* by Arndt *et al*[6]. As these cases were

defined as lacking mutations in sarcomeric genes, the denominator used for *PRDM16* in this cohort was 93 cases (to include the 18 genotype positive cases from the sarcomeric gene studies from this cohort and avoid inflating the frequency of *PRDM16* variants).

See Table S1 for the number of patients sequenced per gene and per cohort.

Defining rare variants

Rare variants were defined as having a filtering allele frequency (FAF) in gnomAD exomes (v 2.1) less than 0.0001. FAFs are defined as the highest disease-specific maximum credible population allele frequency for which the observed allele count is not compatible with pathogenicity. The threshold of 0.0001 was applied in our previous study on rare variant association analysis in cardiomyopathies[7] and has been demonstrated to be an appropriate level based on the specific characteristics (prevalence, genetic heterogeneity and penetrance) in cardiomyopathies[8]. To be defined as rare, variants had to be below this threshold for both the popmax FAF (highest allele frequency of the major sub-populations of gnomAD – African, East Asian, Latino, non-Finnish European and South Asian) and the overall FAF (to filter variants that may be enriched in the gnomAD founder populations – Ashkenazi Jewish and Finnish).

Calculation of denominator for gnomAD

To account for variable coverage in exome-sequenced gnomAD samples, the number of individuals deemed sequenced per gene in gnomAD was calculated using the mean number of alleles across all rare protein-altering variants detected in gnomAD for the gene in question, and then converted from allele number to individuals (divided by 2 for autosomal genes and 1.46 for X chromosome genes based on the male-female ratio in gnomAD).

Specific additional analysis for RBM20, HCN4 and RYR2

In addition to assessing overall gene level rare variant frequencies for truncating and non-truncating variants, further analysis of specific variant classes was performed for three genes (*RBM20*, *HCN4* and *RYR2*) based on previously published reports on the pathogenicity of such variants (described in detail in Table S11). For *RBM20*, non-truncating variants in the established pathogenic hotspot between residues 634 and 638 inclusive were assessed[9,10]. For *HCN4*, non-truncating variants in the transmembrane region of the channel were assessed (amino acid residues 267 to 517 as defined by Uniprot entry Q9Y3Q4).

For *RYR2*, we additionally assessed the frequency of structural variants (SVs) in LVNC cases and controls. As controls, we used the recently added SV dataset in gnomAD, derived from whole genome sequencing of 14,891 individuals[11]. Of note, the detection of SVs requires particular assays and/or analysis pipelines, and it is uncertain whether all of the constituent studies in this analysis were designed to detect such variants. However, we have decided to use the total number of cases where *RYR2* is sequenced (429) to provide a conservative estimate of the contribution of these variants in LVNC.

Cardiomyopathy cohorts

For comparison to the burden of rare variation in dilated cardiomyopathy (DCM) and hypertrophic cardiomyopathy (HCM), variants from cardiomyopathy cases from previously published studies or unpublished data from cardiomyopathy cohorts at the Royal Brompton Hospital were used and re-analysed using the same methods as with the LVNC data. Specifically, previously published data from clinical genetics cohorts from the Oxford Molecular Genetics Laboratory (OMGL), UK, and the Laboratory of Molecular Medicine (LMM), USA was used for *ACTC1*, *ACTN2*, *MYBPC3*, *MYH7*, *TNNT2*, *TPM1* and *TTN* (for DCM) and *ACTC1*, *ACTN2*, *MYBPC3*, *MYH7*, *TNNT2* and *TPM1* (for HCM)[7]. For the remaining genes – *HCN4*, *PRDM16* and *RBM20* (for DCM and HCM) and *TTN* (for HCM), previously unpublished data from cardiomyopathy outpatient clinic cohorts from the Royal Brompton Hospital, UK, was used, comprising up to 863 unrelated DCM patients and up to 685 unrelated HCM patients. See Table S4 for details of the number of DCM and HCM cases analysed for each gene.

Association analysis between genotype and age of onset in LVNC

To assess if there is any significant association between the enriched LVNC variant classes and the age at diagnosis or presentation, we analysed a subset of the case cohort for which this information was available (LMM and Dutch cohorts). Patient ages were dichotomised into adult (≥18 years) and child (<18 years), and the potential over-representation of specific variant classes in either age group was assessed by means of two-sided binomial exact tests, performed with the R function *binom.test()*. Note that for the variants detected in the Dutch cohort, patient age information was only available for those classified as (likely) pathogenic by van Waning *et al*. All VUS from that study were classified as having an unknown age at presentation and excluded from this analysis.

Analysis of the pedigree with the c.732+1G>A variant (index case from the Italian cohort)

The 31-year-old male proband presented at echocardiographic examination with apical and lateral non-compaction of the left ventricle, and marked hypertrabeculation. Of note, he had undergone cardiac ablation at 15 years of age to treat ventricular pre-excitation, and was the only carrier of the p.Pro83Ser variant in *PRKAG2*, which he likely inherited from the father, and which was previously proposed as a genetic modifier predisposing to ventricular pre-excitation[12]. Sequencing was performed on the proband and his family members using the Illumina TruSight Cardio panel.

RNA analysis of MYH7:c.732+1G>A variant

Blood from carriers of the *MYH7*:c.732+1G>A variant in the Italian pedigree was collected in PAXgene Blood RNA Tubes (Preanalytix Cat No.: 762165). RNA was isolated according to the protocol from the kit PAXgene Blood RNA kit (Preanalytix Cat no.: 762174). cDNA was synthesized with the SuperScript™ II Reverse Transcriptase kit (Invitrogen Cat no.: 18064071); whereby the input total RNA was 500ng/sample/reaction. Random hexamers (50pmol/rxn) (Invitrogen Cat no.: N8080127) were used as well as a mix of Random Hexamers (50pmol/rxn) and OligodT-primers (125pmol/rxn) for cDNA synthesis. After synthesis, cDNA was 4x diluted for use in LightCycler480 experiments. Primers used for amplifying *MYH7* transcripts were: *MYH7*_6/7Forw (5'-CTGGATGATCTACACCTACTCG-3') with *MYH7*_int8_9R (5'-CTGGAGAGATGGAAGAGAGTG-3') and *MYH7*_31_32F (5'-GCAGAGGAACTTCGACAAGATC-3') with *MYH7*_34_Rev (5'-CTGCCTTGATCTGGTTGAACTC-3'); 5pmol of

each primer was used per primer-pair/reaction, annealing temperature 57.7 degrees Celsius. We were unable to amplify any detectable *MYH7* product in these samples.

MYH7 truncating variants in population cohorts

Of the UK Biobank individuals with exome sequencing data at the time of analysis (n=49,960 participants), those with cardiac magnetic resonance (CMR) imaging (n=12,447) were included in this study. Individuals with truncating variants (frameshift, nonsense, splice acceptor, splice donor) in *MYH7* were analysed, alongside controls without *MYH7* truncating variants that were matched for year-of-birth, sex, and ethnicity. All individuals were flagged as genetically Caucasian (UK Biobank phenotype ID 22006) and were not related (based on UK Biobank phenotype ID 22021 and KING kinship coefficient estimates from the genotype relatedness file with a cut-off of 0.0884 to include pairs of individuals with greater than 3rd-degree relatedness[13] through ukbtools[14]). *MYH7* truncating variant carriers and year-of-birth-, sex-, and ethnicity-matched non-carrier controls were also identified from healthy control cohorts from the UK Digital Heart Project (n=912)[15] and the Egyptian Collaborative Cardiac Genomics (ECCO-GEN) Project (n=400)[16], all of which had CMR imaging and TruSight Cardio gene panel sequencing[17]. The CMR data of all paired participants was randomised and analysed blindly by two cardiologists who calculated the max NC/C ratio for each individual, and the mean of the ratio from the two separate analyses was assessed. This research has been conducted using the UK Biobank Resource under Application Number 47602.

Power calculations

Prior to the main analysis, we performed power calculations to assess the statistical power provided by the comparison of 840 patients with 120915 population individuals (average number of sequenced gnomAD individuals over the genes we tested). At alpha=0.05, the comparison of 840 cases with 120915 controls provides 80% power to detect an enrichment of 1% against a background variation rate of 0.91% for a single gene tested.

After the analysis, we have re-ran specific power calculations to assess the statistical power we had in those comparisons where - in spite of the enrichment being significant - burdens were representative of extreme cases:

- lowest burden in LVNC cases (non-truncating variants in the *RBM20* hotspot, 3/546 cases vs 1/76260 gnomAD individuals): Cohen's effect size h=0.142, power=95.2%.
- highest background variation rate in gnomAD (*MYBPC3* non-truncating variants, 26/805 cases vs 1780/115675 gnomAD individuals): Cohen's effect size h=0.113, power=93.8%.
- lowest absolute enrichment vs gnomAD (*RBM20* truncating variants, 3/546 cases vs 18/76260 gnomAD individuals): Cohen's effect size h=0.117, power=86.2%.

Power calculations were performed using the *ES.h()* and *pwr.2p2n.test()* functions of the R package pwr.

Supplemental Figures

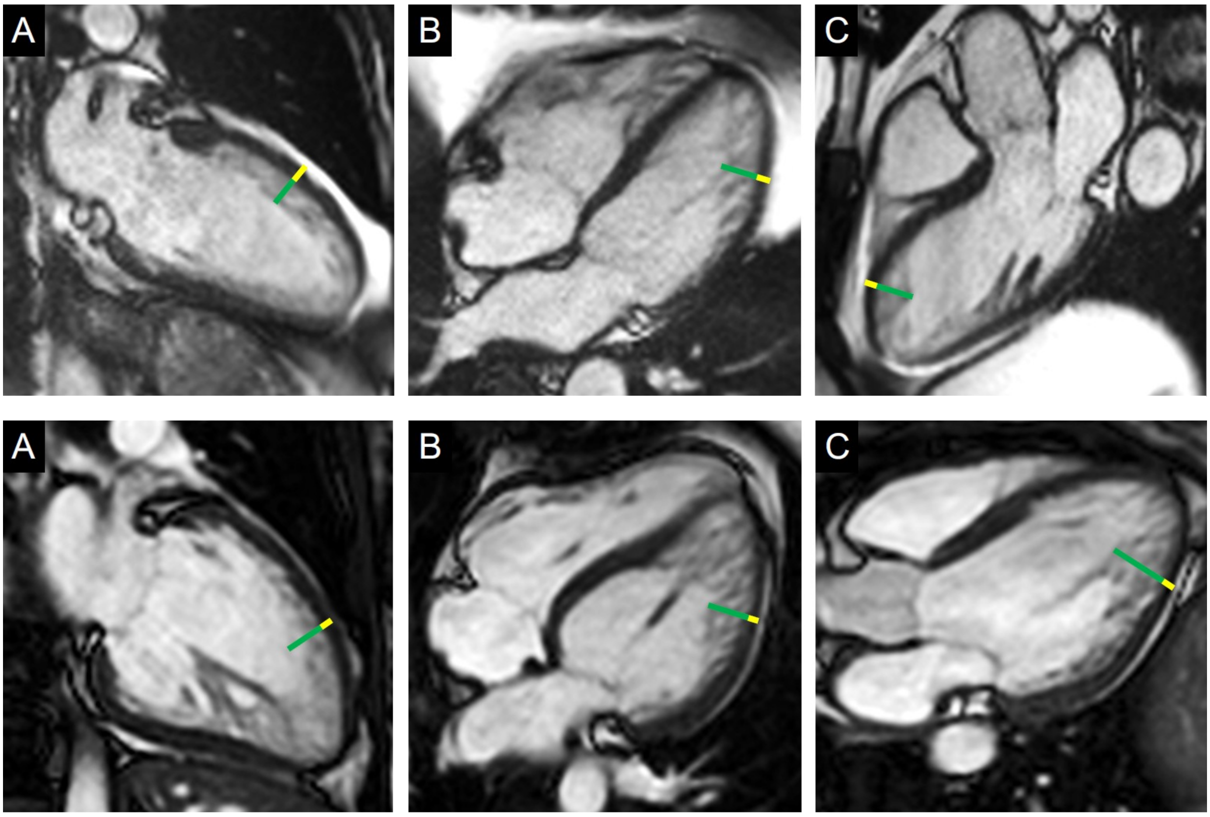

Figure S1: Measurements of non-compacted myocardium (green) and compacted myocardium (yellow) used to calculate NC/C ratio on MR imaging; 2 chamber(A), 4 chamber (B) and 3 chamber (C) projections. Above – UK biobank individual with the MYH7:c.2566_2573delTTCACACGinsCGCG variant. Below - UK Digital Heart Project healthy volunteer with the MYH7:c.733-2A>G variant.

List of Supplemental Tables

Supplemental Table 1: List of genes and Ensembl transcripts analysed in this study (i.e. those analysed in ≥50% of the constituent cohorts). The number of LVNC probands sequenced for each gene in each cohort is shown, along with the total number of cases analysed for each gene in this meta-analysis.

Supplemental Table 2: List of all rare, protein-altering variants detected in the LVNC cases analysed in this meta-analysis for the genes in Table S1. Rarity is defined as a gnomAD exomes (v2.1) filtering allele frequency (overall and popmax) < 0.0001. The variant classes included are frameshift, nonsense and essential splice site (truncating), missense and inframe insertions/deletions (non-truncating) and exon deletions (structural variants). The number of cases in which the variant is detected (per cohort) is also shown. For variants of those classes that are enriched in LVNC, clinical classification information (based on ClinVar v201909) is provided.

Supplemental Table 3: Protein regions ('clusters') where rare non-truncating variants are significantly concentrated in LVNC patients, as detected by the unsupervised clustering algorithm presented in Walsh *et al*, Genome Med, 2019 (PMID 30696458).

Supplemental Table 4: For the genes/variant classes enriched in LVNC, we compared rare variant frequencies and enrichment over gnomAD population controls in dilated cardiomyopathy (DCM) and hypertrophic cardiomyopathy (HCM) cohorts described here. OMGL/LMM indicates data from clinical genetics cohorts from the Oxford Molecular Genetics Laboratory (UK) and the Laboratory of Molecular Medicine (Boston, USA), previously published by Walsh *et al* (Genet. Med. 2017). Royal Brompton indicates prospective cohorts from outpatient clinics at the Royal Brompton Hospital (London, UK), with up to 863 DCM probands and up to 685 HCM probands sequenced on the TruSight Cardio panel.

Supplemental Table 5: Results for rare variant burden testing in LVNC cases versus gnomAD exomes (v2.1) population controls. Data for truncating and non-truncating variants in 70 genes are shown, as well non-truncating variants within and without the *HCN4* transmembrane region and the *RBM20* pathogenic hotspot and structural variants (exon deletions) in *RYR2* (compared to the gnomAD structural variants dataset). Variant counts and rare variant frequencies for LVNC and gnomAD are displayed, as well as the excess of rare variants in LVNC and the p value for the Fisher's exact test. Note - *TTN* non-truncating variants were not reported in the Miszalski-Jamka et al cohort and therefore there are different LVNC sample numbers for *TTN* truncating and non-truncating variants.

Supplemental Table 6: Assessment of enrichment of rare variants is LVNC-associated variant classes in either adult probands (≥ 18 years) or children (<18 years), by means of two-sided binomial exact tests performed with the R function *binom.test()*. Analysis is based on cases in the LMM cohort (95 adults and 138 children) and the Dutch cohort (275 adults and 52 children). Note, in the Dutch cohort ages were only available for those probands with a (likely) pathogenic variant as defined by van Waning *et al*.

Supplemental Table 7: Frequencies of rare variants in each of the constituent LVNC cohorts for the variants classes significantly enriched in LVNC compared to gnomAD exomes population controls.

Supplemental Table 8: Frequency details for the common *MYH7* splice region variant, c.732C>T, in the newly described LVNC cohorts of this study and the appropriate gnomAD sub-populations. P values are from a two tailed Fisher's exact test. Ethnicities for the LMM cases are self-declared.

Supplemental Table 9: List of *MYH7* truncating variants (described with reference to the ENST00000355349 transcript) detected in 12,447 individuals in UK Biobank (individuals with both exome sequencing and CMR imaging), 912 healthy controls from the UK Digital Heart Project and 400

healthy controls from the Egyptian Collaborative Cardiac Genomics (ECCO-GEN) Project. NC/C refers to the maximum ratio of non-compacted to compacted left ventricular myocardium (values are the mean of independent measurements from two cardiologists). NC/C ratios of year-of-birth-, sex-, and ethnicity-matched non-carriers from the respective cohorts are also displayed. * Variant was described in UK Biobank data as two adjacent variants.

Supplemental Table 10: Details of the LVNC cases with rare variants in the genes/variant classes that are enriched in LVNC but not enriched in DCM or HCM – *ACTN2* truncating variants, *HCN4* missense variants in the transmembrane region, *PRDM16* truncating variants and *RYR2* exon deletions. See table 3 in the manuscript for details LVNC cases with *MYH7* truncating variants.

Supplemental Table 11: Summary of published studies supporting a role in the non-compaction phenotype for *PRDM16* and *ACTN2* and in arrhythmogenic and non-compaction phenotypes for *RYR2* and *HCN4*.

Supplemental Table 12: Recommendations for rare variant interpretation using the ACMG/AMP guidelines for patients with LVNC cardiomyopathy. As patients with LVNC may have underlying DCM or HCM, variants in known DCM/HCM genes/variant classes should be interpreted according to standard DCM/HCM approaches, with adaptations for *MYH7* and *ACTC1* non-truncating variants as described below. ACMG/AMP rules for LVNC-specific variant classes: PVS1 (loss-of-function truncating variants) to be applied according to recommendations of Tayoun *et al* (Human Mutation 2018;39:1517–1524); PM1 (non-truncating variant hotspots) applied according to methods described by Walsh *et al* (Genome Med. 2019;11(1):5) based on etiological fraction (EF) values derived from case-control analysis of rare variants. Other standard ACMG/AMP rules should be applied as appropriate.

Supplemental References

1. van Waning JI, Caliskan K, Hoedemaekers YM, et al. Genetics, Clinical Features, and Long-Term Outcome of Noncompaction Cardiomyopathy. *J Am Coll Cardiol*. 2018;71(7):711-722. doi:10.1016/j.jacc.2017.12.019

2. Richard P, Ader F, Roux M, et al. Targeted panel sequencing in adult patients with left ventricular non-compaction reveals a large genetic heterogeneity. *Clin Genet*. 2019;95(3):356-367. doi:10.1111/cge.13484

3. Miszalski-Jamka K, Jefferies JL, Mazur W, et al. Novel Genetic Triggers and Genotype-Phenotype Correlations in Patients With Left Ventricular Noncompaction. *Circ Cardiovasc Genet*. 2017;10(4). doi:10.1161/CIRCGENETICS.117.001763

4. Klaassen S, Probst S, Oechslin E, et al. Mutations in sarcomere protein genes in left ventricular noncompaction. *Circulation*. 2008;117(22):2893-2901. doi:10.1161/CIRCULATIONAHA.107.746164

5. Probst S, Oechslin E, Schuler P, et al. Sarcomere gene mutations in isolated left ventricular noncompaction cardiomyopathy do not predict clinical phenotype. *Circ Cardiovasc Genet*. 2011;4(4):367-374. doi:10.1161/CIRCGENETICS.110.959270

6. Arndt A-K, Schafer S, Drenckhahn J-D, et al. Fine mapping of the 1p36 deletion syndrome identifies mutation of PRDM16 as a cause of cardiomyopathy. *Am J Hum Genet*. 2013;93(1):67-77. doi:10.1016/j.ajhg.2013.05.015

7. Walsh R, Thomson KL, Ware JS, et al. Reassessment of Mendelian gene pathogenicity using 7,855 cardiomyopathy cases and 60,706 reference samples. *Genet Med*. 2017;19(2):192-203. doi:10.1038/gim.2016.90

8. Whiffin N, Minikel E, Walsh R, et al. Using high-resolution variant frequencies to empower clinical genome interpretation. *Genet Med*. 2017;19(10):1151-1158. doi:10.1038/gim.2017.26

9. Brauch KM, Karst ML, Herron KJ, et al. Mutations in ribonucleic acid binding protein gene cause familial dilated cardiomyopathy. *J Am Coll Cardiol*. 2009;54(10):930-941. doi:10.1016/j.jacc.2009.05.038

10. Li D, Morales A, Gonzalez-Quintana J, et al. Identification of novel mutations in RBM20 in patients with dilated cardiomyopathy. *Clin Transl Sci*. 2010;3(3):90-97. doi:10.1111/j.1752-8062.2010.00198.x

11. Collins RL, Brand H, Karczewski KJ, et al. A structural variation reference for medical and population genetics. *Nature*. 2020;581(7809):444-451. doi:10.1038/s41586-020-2287-8

12. Scheffold T, Waldmüller S, Borisov K. A case of familial hypertrophic cardiomyopathy emphasizes the importance of parallel screening of multiple disease genes. *Clin Res Cardiol*. 2011;100(7):627-628. doi:10.1007/s00392-011-0306-3

13. Manichaikul A, Mychaleckyj JC, Rich SS, Daly K, Sale M, Chen WM. Robust relationship inference in genome-wide association studies. *Bioinformatics*. 2010;26(22):2867-2873. doi:10.1093/bioinformatics/btq559

14. Hanscombe K, Coleman JR., Traylor M, Lewis C. ukbtools: An R package to manage and query UK Biobank data. *PLoS One*. 2019;14(5):e0214311. doi:10.1101/158113

15. de Marvao A, Dawes TJW, Shi W, et al. Precursors of Hypertensive Heart Phenotype Develop in Healthy Adults: A High-Resolution 3D MRI Study. *JACC Cardiovasc Imaging*. 2015;8(11):1260-1269. doi:10.1016/j.jcmg.2015.08.007

16. Aguib Y, Allouba M, Afify A, et al. The Egyptian Collaborative Cardiac Genomics (ECCO-GEN) Project: defining a healthy volunteer cohort. *NPJ genomic Med*. 2020;5:46. doi:10.1038/s41525-020-00153-w

17. Pua CJ, Bhalshankar J, Miao K, et al. Development of a Comprehensive Sequencing Assay for Inherited Cardiac Condition Genes. *J Cardiovasc Transl Res*. 2016;9(1):3-11. doi:10.1007/s12265-016-9673-5
